# Perception and Awareness of Animal Welfare Among Residents of Malta †

**DOI:** 10.3390/ani15111634

**Published:** 2025-06-02

**Authors:** Pantaleo Gemma, Eleonora Nannoni, Barbara Padalino, Angelo Peli, Francesco Luca Alexander, Giovanni Buonaiuto, Luca Sardi, Giovanna Martelli

**Affiliations:** 1 Department of Agricultural and Food Sciences, University of Bologna, 40126 Bologna, Italy; pantaleo.gemma2@unibo.it (P.G.); barbara.padalino@unibo.it (B.P.); 2 Department of Veterinary Medical Sciences, University of Bologna, 40064 Ozzano Emilia (BO), Italy; giovanni.buonaiuto@unibo.it (G.B.); luca.sardi@unibo.it (L.S.); giovanna.martelli@unibo.it (G.M.); 3 Department for Life Quality Studies, University of Bologna, 47921 Rimimi, Italy; angelo.peli@unibo.it; 4 Department of Rural Sciences and Food Systems, University of Malta, 2080 Msida, Malta; francesco.alexander.19@um.edu.mt

**Keywords:** Malta, consumer behaviour, willingness to pay, animal-friendly products

## Abstract

A representative sample of Maltese citizens (N = 384) was surveyed about their perception and attitude towards animal welfare and animal-friendly foods. Knowledge about animal welfare was self-evaluated as moderate (36%) or good (27%), and mass media (television, web and newspapers) were the primary information source (73%). Dairy cows were perceived as having the highest welfare (average rating 3 on a 1-to-5 scale), while conditions for broilers and pigs were perceived as more critical (average rating 2.7). Respondents consider animal welfare important (64%), the availability of welfare-friendly products in Malta limited (49%), and would support a national animal-friendly label (84%). Although 49% were willing to pay more for animal-friendly products, the accepted price increase was limited (increase below 10% for 37% of respondents, 20% were not willing to pay more and 27% were price-sensitive). Chi-squared analysis showed that the respondents’ profession impacted the support towards an animal welfare label (*p* < 0.01), with business operators being the least interested. Urban respondents were more critical toward farm animal welfare and more supportive of establishing a national welfare label than rural respondents (*p* < 0.05). The latter were less convinced that their choices can influence the welfare of farm animals (*p* < 0.01). Women were more willing to pay for welfare improvements than men (*p* < 0.01). These findings emphasize a significant concern for animal welfare among respondents, coupled with a moderate willingness to pay. The strong support for a national animal welfare label warrants the question as to whether transparent labelling would allow consumers to translate their sensibility into ethical purchasing behaviour.

## Simple Summary

This study investigated consumer perceptions and purchasing behaviour regarding animal welfare and animal-friendly foods in Malta. Respondents expressed different perceptions about the farming conditions of different species, with dairy cow welfare levels seen as the highest and broilers and pigs perceived as the lowest. Overall, the animal welfare knowledge level was moderate, with mass media (television, web, newspapers) serving as the primary information source. Most respondents considered animal welfare in food purchasing decisions, and there was broad support for welfare certification labels, including strong support (84%) for a national welfare label. Willingness to pay more for higher welfare standards was common, although the price increase accepted was generally low (below 10%). The availability of welfare-friendly products in Malta was perceived as limited (49% of respondents). Occupation, urban residence, and gender influenced animal welfare perception and support. These findings underline a potential role for welfare labels and transparent communication to support ethical purchasing behaviours, with implications for future policy improvements and consumer information.

## 1. Introduction

The demand for food of animal origin is increasing worldwide as a consequence of global population growth [1,2]. Considering that animal welfare has a positive impact on productivity and on the safety and quality of animal-derived foods [3,4,5], the importance of animal welfare in food production has gained increasing attention, reflecting broader societal concerns regarding ethical consumption and sustainable agricultural practices [6,7,8,9]. Furthermore, national and international organizations such as the World Organisation for Animal Health (WOAH) highlighted the strong links between human and animal welfare and health and their role in the sustainability of both socioeconomic and ecological systems [10], suggesting a One Health, One Welfare approach [1]. Understanding consumer attitudes towards animal welfare is essential for various stakeholders, including policymakers, retailers, and producers, as it can guide initiatives to promote more humane and sustainable food production practices [11,12] while meeting consumers’ ethical expectations. Highlighting the welfare and One Health content could allow farmers to set their products apart and improve their competitiveness [13].

Animal welfare has progressively emerged as a prominent policy issue within the European Union (EU) [14]. Over the years, EU institutions have promoted several Eurobarometers (large, cross-country public opinion surveys conducted regularly on strategic topics for the EU) focusing on animal welfare perception by European citizens. The adoption of the Farm to Fork Strategy in 2020 marked a significant step towards transforming the European food system, emphasizing sustainability and ethical considerations in food production [15]. A critical component of this strategy is the commitment to update existing animal welfare laws and regulations, including the discussion regarding the introduction of a European Animal Welfare label.

The European Commission is, therefore, exploring the potential implementation of a harmonized animal welfare labelling system. Such a system would serve multiple purposes; it would provide European citizens with a consistent framework for understanding animal welfare standards, thereby promoting transparency in the market. By having clear and accessible information, consumers would be able to make informed choices about the food they buy, aligning their purchases with their ethical beliefs regarding animal welfare [16].

Furthermore, an EU-wide animal welfare label could have broader implications for production standards across Member States. By elevating the requirements for animal welfare, it could stimulate producers to adopt higher welfare standards, ultimately leading to improved farming conditions (animal-friendly foods). Additionally, a unified labelling system would help mitigate the challenges associated with unfair trade practices, as it could deter the importation of products from countries with substandard animal welfare regulations [17]. In doing so, the proposed labelling initiative not only aims to protect consumer interests but also seeks to promote ethical and sustainable food production practices within the European Union [18,19].

Malta currently lacks a standardized animal welfare labelling system, which poses significant challenges for consumers in a nation characterized by high population density and a limited capacity for domestic production of animal-derived foods. As a result, a substantial proportion of these products on the Maltese market is sourced from other European and third countries, which may adhere to differing animal welfare standards. This discrepancy can create confusion and uncertainty among Maltese consumers regarding the welfare conditions associated with the products they purchase.

In light of the interplay between various local factors, such as the island’s small size, the significance of tourism, the growing development of the Information Technology (IT) industry, and the strong cultural ties to rural traditions, and external influences, including the legacy of British rule and Malta’s membership in the European Union, this study aimed to investigate current consumer perceptions and knowledge regarding animal welfare and animal-friendly foods in Malta, as well as their impact on purchasing behaviour. These peculiarities pushed us to carry out a survey, partly based on the recent Eurobarometers’ questions and partly in order to explore poorly known aspects of Maltese consumers’ perception of animal welfare (e.g., consumption habits, perceived welfare level of farmed animals, degree of trust in a national label, effects of demographic characteristics).

## 2. Materials and Methods

All procedures used in this study received approval from the University of Bologna’s Bioethics Committee (protocol number 0289930 on 9 October 2023).

### 2.1. Questionnaire

A structured questionnaire was prepared, based on previous studies [18,19] and Eurobarometer surveys [20,21,22]. The questionnaire consisted of 23 questions, part closed-ended and part Likert scales, and can be found in the Supplementary Materials. It addressed the following themes:

- Sociodemographic information and purchasing habits: This section gathered data on respondents’ demographics (rural or urban area, gender, age, education level, income, and household size). It also asked about the frequency and types of meat consumed.
- Knowledge of animal welfare: Respondents rated their perceived level of knowledge regarding animal welfare and listed the sources from which they obtained this knowledge. This section also investigated the perceived effects on animal welfare of some farming aspects (e.g., sufficient space, humane slaughtering) and knowledge about animal protection legislation.
- Perception of animal welfare: Participants were asked to express how they perceived the welfare level of several farm animal species.
- Purchasing behaviour: This last part asked questions about purchasing decisions, including the importance of animal welfare when buying animal products and the perceived need and specific expectations about animal welfare labelling.

Once the questionnaire was ready, a pilot test was run with a group of colleagues from the Animal Science section of the Department of Veterinary Medical Sciences (DIMEVET) of the University of Bologna, to identify possible unclarities in the questionnaire formulation and to have an approximate compilation time.

### 2.2. Questionnaire Administration

The structured questionnaire was strictly followed to carry out a telephone survey in Malta, from the 1 February 2024 until the 31 May 2024. Phone calls were carried out by retrieving publicly available phone numbers using an online telephone database and sampling a representative percentage of participants from various towns across Malta. The total number of 384 respondents was decided based on the power analysis performed on the Maltese population (that, in 2023, was 532,956 inhabitants [23]) with a 95% Confidence Interval and a 5% margin of error. Phone calls were carried out by two of the co-authors from Monday to Sunday between 15:00 and 20:00 since it was the most convenient time for the respondents. The language used was either Maltese or English depending on the preference of the respondent. At the beginning of the call, consumers were informed about the aim of the research and the questionnaire topic, and their oral consent to participate was obtained before beginning the survey. A total of 384 responses were obtained. The response rate was 88.5%, as 50 consumers (50/434, i.e., 11.5%) refused to be surveyed, mostly due to lack of time.

### 2.3. Data Analysis

Data were transferred to an Excel spreadsheet to obtain a matrix of the responses. Descriptive statistics (percentages, average scores) of the answers were calculated.

Chi-squared statistics or Fisher’s exact tests were then calculated using the software Jamovi 2.3.28 [24], to assess the effects of the demographic characteristics (gender, age, household size, education, job, household income, and urban or rural residence) of the respondents on the main responses (self-perception of animal welfare knowledge, importance attributed to animal welfare, perceived animal welfare level of animals farmed in Malta, willingness to pay more for animal-friendly foods, support of a national welfare label, and perceived effect of purchasing choices on the welfare of farm animals). For each question, the analysis was carried out in two-way contingency tables reporting the number of observations (i.e., the frequencies) for each possible answer and possible level of the demographic characteristic. When the contingency tables contained values below 5 in any of the cells, Fisher’s exact test was used, and the *p*-value was estimated based on 10,000 Monte Carlo permutations. Only statistically significant class distributions are reported. When class distributions statistically differed, pairwise comparisons were carried out using either Chi-squared tests or Fisher’s exact test (with the same criteria described above).

A cluster analysis was also conducted using the k-means algorithm, implemented in Python 3.13.3, on all variables included in the dataset. The optimal number of clusters was determined using the inertia index (within-cluster sum of squares) and identifying the inflection point in the elbow plot. Based on this evaluation, three clusters were selected. The k-means algorithm was then run with the number of clusters set to three. To facilitate the visualization of the clustering results, Principal Component Analysis (PCA) was performed on the clustered data, and the two-dimensional output was used to generate a graphical representation.

The threshold for statistical significance was set at *p* < 0.05 for all tests.

## 3. Results

### 3.1. Descriptive Analysis

Sociodemographic information is presented in Table 1. The present survey gathered data from a balanced gender distribution. The largest share of respondents was in the 25–39 age group (38%). The education level showed almost 40% of respondents holding tertiary degrees (degree provided by University of Malta and the Malta College of Arts, Science and Technology (MCAST) students that are older than 18 years old), followed by post-secondary qualifications (upper secondary school between 16 and 18 years of age or vocational school between 16 and 20 years). Most participants (73%) lived in urban areas and were employees (67%). Household size varied from one to eight people, but most respondents came from households of three or four people (57%). The most common annual income range was between EUR 21,000 and EUR 50,000 (40%), although one-third of respondents preferred not to disclose income data.

Regarding consumption habits, the vast majority (92%) of respondents reported following an omnivorous diet. Chicken and beef emerged as the most frequently consumed meats, with 50% of the respondents eating chicken and 21% eating beef more than once a week. Fish and chicken were commonly consumed at least once a week by 35% and 38% of respondents, respectively. Rabbit and pork were typically eaten at least once a month (28 and 19% of respondents), and lamb was consumed monthly by 24% of the respondents. Lamb and rabbit were the least consumed meats, with 31% and 22% of respondents reporting never eating them.

Most respondents perceived their knowledge of animal welfare as medium (36%) or good (27%), while 9% perceived themselves as having very good knowledge, and 3% reported having only basic knowledge. Mass media (television, web, newspapers) were the predominant source of information on animal welfare (73%), followed by direct knowledge (farm visits, 13%) and by people having a farming, agronomical, or veterinarian occupation (11%). Eleven respondents (3%) reported school as the main source of information.

**Figure 1:**
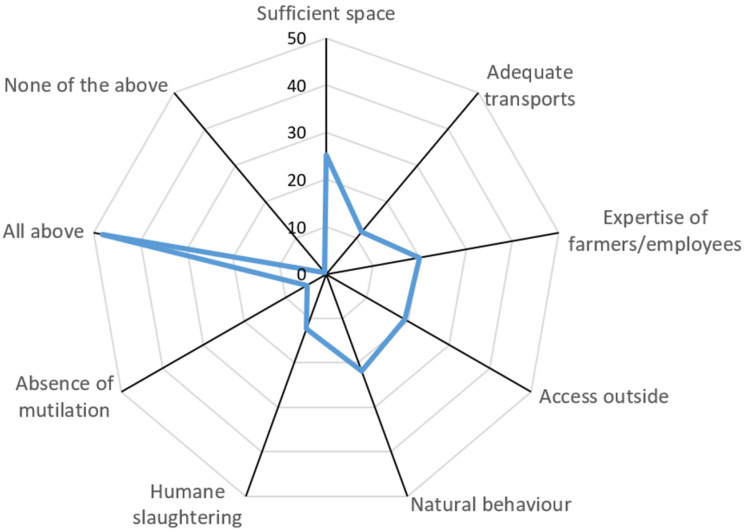
Radar chart of the answers to the question “In your opinion, which ones, among these aspects, are the most important in determining the level of animal welfare?” (N = 384 respondents, multiple answers allowed). The nine angles represent the available response options, and the numbers represent the percentage of respondents who selected each one.

When asked about the most important factors in determining animal welfare (multiple answers were allowed), 25% of respondents selected sufficient space, 21% natural behaviour, 20% expertise of the farmer or employees, 19% access outside, 12% humane slaughter, 11% adequate transport, 5% absence of mutilation, and 48% of respondents believed all the mentioned aspects to be important. Results are shown in Figure 1.

When asked which of the production phases are regulated by laws on animal protection in Malta (multiple answers allowed), the majority (52%) answered that all phases (slaughter, feeding, rearing and transport) are regulated, 25% selected slaughtering, 17% feeding, 13% rearing, 11% transport and 4% none of the above.

**Figure 2:**
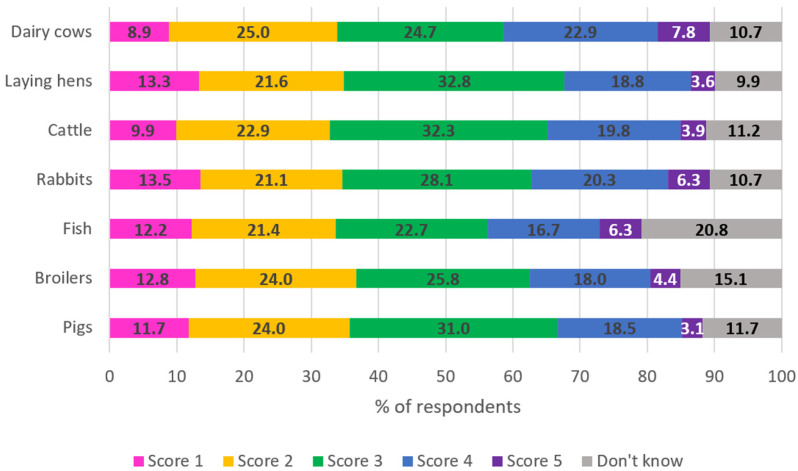
Answers (N = 384) to the question “Please rate on a 1-to-5 scale to the level of welfare you think the following species have on farm (1 = poor; 2 = just acceptable; 3 = medium; 4 = good; 5 = excellent)”. The numbers represent the percentage of answers per each score.

Figure 2 shows answers to the perceived welfare level of various farmed animal species on a 1-to-5 scale, where 1 represented the lowest perceived welfare and 5 the highest perceived welfare. Responses are shown in Figure 2.

In summary, dairy cows’ welfare received the highest average scores (3, indicating a medium welfare level). Rabbits, laying hens, and cattle had an intermediate score (average score: 2.8 for each of the three species). Broilers and pigs’ welfare levels were more frequently scored as lower, resulting in the most critical perceived welfare level (average score is 2.7 for both species). Lastly, the fish welfare level was unknown by several respondents, while the remaining ones judged it as moderate (average score: 2.8). Overall, the average scores were very similar across species.

Respondents were also asked to score, on a 1–5 scale, the importance they attribute to animal welfare at the time of food purchasing. Thus, 10% consider it very important (rating 5), 15% consider it quite important (rating 4), and 40% assign an intermediate rating (rating 3). The remaining consumers find it of little importance (rating 2, 27%) or not important at all (rating 1, 9%).

When asked to choose from a list what products are obtained respecting higher animal welfare standards (multiple answers were allowed), a majority of respondents (63%) answered certified animal-friendly labels. This was followed by organic products (49% of respondents), extensive farming (23%), Protected Designation of Origin (PDO) products (10%) and intensive farming (7%).

**Figure 3:**
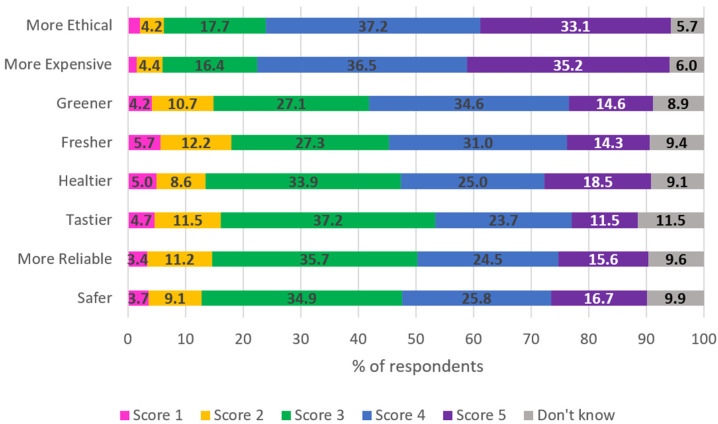
Answers (N = 384) to the question: “In your opinion, products obtained respecting high animal welfare standards are also: (1 = completely disagree; 2 = partially disagree; 3 = neutral; 4 = agree; 5 = completely agree)” The numbers represent the percentage of answers per each score.

With respect to characteristics attributed by respondents to animal-friendly foods (rating 4 or 5 on a 5-point scale), 70% of respondents saw these products as more ethical, 71% as more expensive, 50% as greener, 43% as safer, 45% as fresher, and 43% as healthier that conventional foods, 35% tastier and 40% more reliable. A breakdown of the scores for each perceived characteristic is shown in Figure 3.

Respondents’ perception of the welfare level of animals raised in Malta showed that 36% of consumers perceived welfare levels to be medium, 29% just acceptable, 16% good, 11% poor, and 2% excellent. The remaining 6% did not know.

Respondents were also asked about the availability of animal-friendly products in Maltese markets and supermarkets. One half (49%) considered it to be (probably or certainly) insufficient, 36% (probably or certainly) sufficient and 13% answered “I don’t know”.

**Figure 4:**
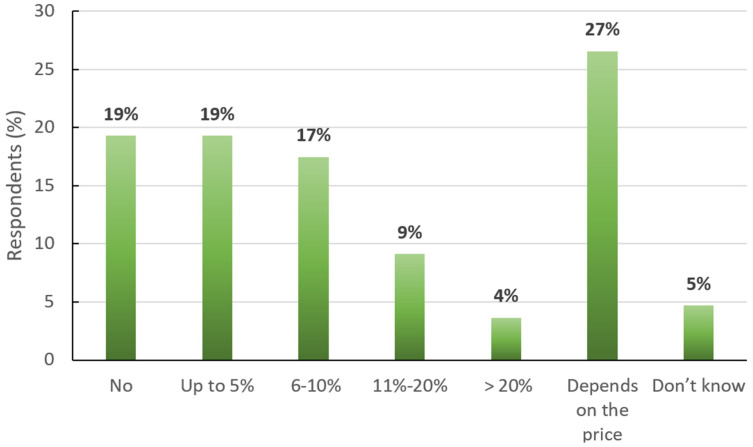
Answers (N = 384) to the question “Would you be willing to pay more for products sourced from animal welfare-friendly (higher animal welfare level) production systems?”. The numbers represent the percentage of respondents who chose each answer.

Respondents were then asked if they would be willing to pay more for products sourced from animal-friendly production systems. Nearly half of the respondents (49%) indicated a willingness to pay more for higher-welfare products, with an accepted price increase up to either 5% or 10% in most cases, as shown in Figure 4. However, the remaining half of consumers were either not willing to pay more or declared that they were price-sensitive, answering that their willingness depended on the cost of the product.

When asked whether they were in favour of a national label indicating the level of welfare of the animals, 84% of respondents expressed support, while 7% were not in favour and 9% did not know.

Respondents also specified the information they would like or expect to find on an animal welfare label. Among the proposed answers (multiple selections were allowed), 25% of respondents selected farm conditions and methods, 4% transport, 4% feeding, 4% use of antibiotics, 3% slaughtering, 1% expertise of the staff and most respondents (59%) selected the answer “all the above”. The remaining 1% chose “none of the above”.

Lastly, respondents were asked whether they believed their purchasing choices as consumers could positively affect animal welfare levels in food production. The majority (49%) responded “Yes, probably”, while 29% answered “Yes, certainly”. On the other hand, 10% said “No, probably not”, 9% were unsure, and 3% replied “No, certainly not”.

### 3.2. Class Distribution Analysis

Table 2 summarizes the significant effects of the demographic characteristics on the answers to selected questions. Occupation affected support for a national animal-friendly label (*p* = 0.006), with students, homemakers and employees being more interested than business owners, retired and unoccupied consumers.

Living in an urban vs. rural area affected (1) the perceived animal welfare level of production animals farmed in Malta (*p* = 0.034), with 14% of urban citizens vs. 28% of rural citizens scoring it as either good or excellent; (2) the support towards a national welfare label (*p* = 0.013, with urban citizens more in favour of the label compared to rural ones); and (3) the perception of how consumer choices may affect the welfare of farm animals (*p* < 0.001, with urban consumers believing more positive than rural ones about the effects of their purchasing behaviour on the welfare level on farms).

Lastly, gender affected willingness to pay (*p* = 0.006): overall, women were more willing to pay than men, particularly when the price increase was low (between 6 and 10%).

### 3.3. Cluster Analysis

**Figure 5:**
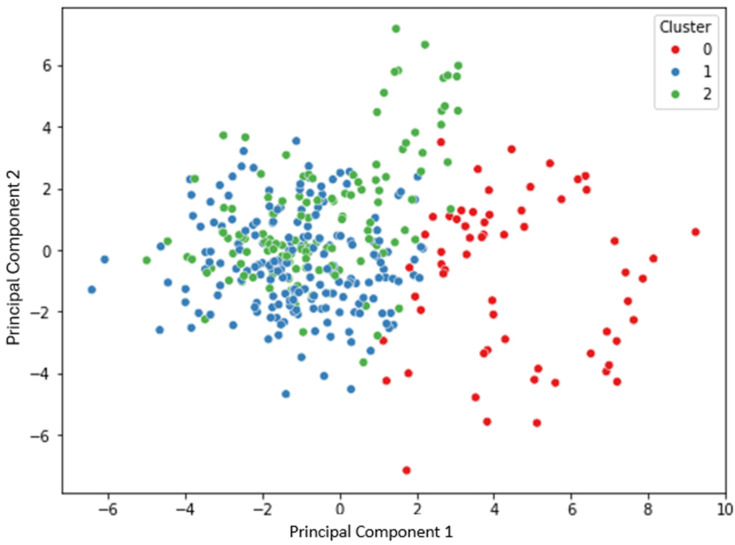
Principal Component Analysis (PCA) plot of the k-means clustering results based on all survey variables. The three clusters identified (Cluster 0 = red, Cluster 1 = blue, Cluster 2 = green) are projected onto the first two principal components.

Cluster analysis based on all variables in the dataset identified three distinct clusters, as determined by the elbow method applied to the inertia index. The results of the k-means clustering were projected onto a two-dimensional space using Principal Component Analysis (PCA), as shown in Figure 5.

While the three clusters (Cluster 0 in red, Cluster 1 in blue, and Cluster 2 in green) can be visually distinguished to some extent, the projection reveals considerable overlap between Cluster 1 and Cluster 2, indicating partial separation in the reduced dimensional space.

## 4. Discussion

### 4.1. Descriptive Analysis

The demographic characteristics of the sample, overall, align well with Malta’s general population, with slightly higher education and income levels when compared to the wider national trends [25,26]. Moreover, they are similar to those reported in previous studies on Italian consumers [18,19].

Consumption habits generally agree with previous findings, although in a previous study, people living in a rural area in Malta showed a higher preference for rabbit, with only 8% of respondents not consuming rabbit meat, with its consumption frequency being in third place after chicken and beef, and rabbit being deeply rooted in the local gastronomic culture [27].

Similarly to previous studies in Italy [18,19], mass media were the primary information source on animal welfare, followed by experiential knowledge such as farm visits and insights from occupational exposure (farmers, agronomists, veterinarians, or occupations in the animal production field).

The heavy reliance on mass media as the primary source of information about animal welfare may leave consumers exposed to potentially biased external information [28]. Studies have shown that mass media, while influential, often provide a fragmented and sensationalistic portrayal of animal welfare issues, which may not fully reflect the complexities of farming practices and animal care [18]. Consequently, consumer perceptions may be shaped more by media narratives than by technical information or direct experience. The limited experience of agricultural environments suggests a disconnection between consumer attitudes and the reality of animal welfare on farms. Similar results in terms of sources of information have been observed in Italy [18]. In this respect, targeted educational campaigns and school education about animal welfare could increase knowledge and awareness, particularly in urban areas where exposure to farming practices is limited.

As concerns the factors mostly affecting animal welfare levels, nearly half of the respondents considered all the listed factors as important to animal welfare. This suggests a holistic understanding of the multifaceted needs of farm animals. In terms of species, respondents expressed concerns about farming practices in those species (pigs and broilers) perceived as kept more intensively, while they seemed to find more acceptable the conditions in which dairy cows are kept. This aligns with previous findings and might suggest priority interventions [6,19,22,29,30]. However, this also warrants the possible misperception by citizens of a farming system, such as the typical dairy one, which, despite usually allowing at least partial access outdoors, is highly intensive and not yet subjected to specific, vertical regulations.

With respect to knowledge about regulations, overall responses indicated a general awareness among respondents of the existence of at least some regulations on animal protection. This may also indicate some level of consumer trust in national and European regulations. However, as demonstrated in previous studies, the fact that only half of the respondents correctly recognize the existence of these regulations for all the production phases suggests a need for increased transparency and communication to help clarify the regulatory frameworks and help in building consumer trust and increasing their ability to make informed choices [19,22,29].

Maltese consumers are quite concerned about animal welfare. The Eurobarometer 2023 [22] reported that over 94% of Maltese respondents believe it is important to protect the welfare of farm animals; however, our findings show that only 64% of respondents pay attention to animal welfare when purchasing food. This is in line with a 2010 report from the European Animal Welfare Platform, where data collected from the UK and six other countries were reported [31].

This highlights a gap between consumers’ interest in ethical issues and their willingness to act on them. While they may value ethical considerations in principle, they are often less likely to make concrete purchasing decisions based on these values. This may be due to factors such as cost, convenience, or doubts about the effectiveness of individual actions [7,32,33].

In terms of recognition of animal-friendly products, consumers did not excel, with only 63% of respondents recognizing animal welfare-certified labels, around 50% organic products and 23% products from extensive farming. Smaller, but not minor, percentages erroneously attributed high animal welfare content to products from intensive farming (6%) or PDO (Protected Designation of Origin) certifications, which typically do not include provisions for animal welfare protection that go beyond the minimum requirements of the legislation. Previous studies showed similar results regarding the PDO products [18,19]. While this suggests certain trust in specific certification programs and organic labelling as indicators of ethical practices, it also shows the presence of misperceptions in the products’ ethical content [18]. On the other hand, one-half of consumers correctly recognized organic products despite the fact that labelling and marketing campaigns for these foods may not specifically focus on animal welfare [18].

Most Maltese consumers associate high animal welfare standards with ethics and higher prices. Surprisingly, many consumers attributed characteristics to these products that are not necessarily related to higher animal welfare content, such as safety, freshness, healthiness, taste and reliability (about 40% for each attribute). These results, similar to other studies, emphasize the presence of unrealistic expectations and the need for transparent communication on the possible positive attributes of animal-friendly foods [34,35]. The animal welfare level in Malta was perceived as only acceptable or medium by most consumers. This is similar to the results in the Eurobarometer 2015 and 2023 [21,22], where 73% and 90%, respectively, of Maltese consumers agreed that farm animal welfare in their country should be improved. Improving welfare standards and effectively communicating the achievements could help shift public perception towards more favourable views of animal welfare practices [5,34].

Less than 40% considered the availability of animal-friendly foods to be sufficient, and one-half considered it insufficient, which may indicate potential for market expansion for these products. Based on data from the Eurobarometer surveys of 2015 and 2023 [21,22], the percentage of consumers perceiving the range of available options for animal-friendly foods as sufficient increased over time, rising from 38% in 2015 to 48% in 2023. In Malta, this perception remains close to the 2015 Eurobarometer data. This may suggest a slower adoption of these labels within the Maltese market.

The perceived lack of availability of high animal welfare products poses a substantial barrier to ethical consumerism, not only in Malta but also in other countries [19,36], as limited options can discourage consumers from making animal-friendly purchasing decisions. Retailers and producers, thus, have a significant opportunity to address this gap by expanding the range of welfare-friendly products available in the market. Furthermore, the current absence of harmonized animal welfare labelling in Malta complicates matters, as products imported from other European and third countries often carry voluntary and varied animal welfare labels. This lack of harmonization leads to confusion among consumers regarding the welfare standards of the products they purchase, hindering their ability to make informed choices and undermining their commitment to ethical consumption [17]. Addressing these issues through the establishment of a cohesive and transparent animal welfare labelling system could empower consumers in Europe, facilitate better market alignment with ethical demands, and enhance the overall integrity of the food supply chain [37,38] in a One Health, One Welfare perspective. Regarding consumers’ willingness to spend more in favour of a higher level of animal welfare, data from the Eurobarometer surveys [21,22] and the present study highlight evolving consumer attitudes toward paying more for animal welfare-friendly products.

**Figure 6:**
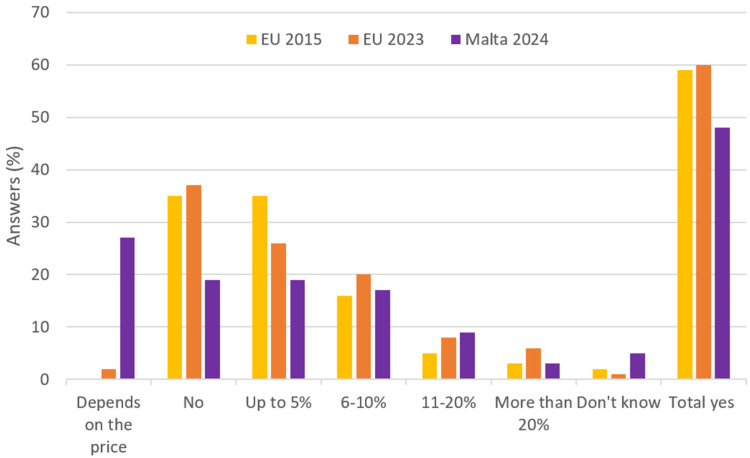
Comparison of the willingness to pay expressed by European consumers in the 2015 Eurobarometer [21], in the 2023 Eurobarometer [22] and in the present study (as reported also in Figure 4).

Figure 6 compares the willingness to pay declared by consumers in the 2015 and 2023 Eurobarometers [21,22] with the results from the current study. In 2024, Maltese consumers gave fewer “yes” or “no” answers and, for several consumers, the willingness to pay was price dependent. When comparing the declared willingness to pay of Maltese consumers to previous Eurobarometers [21,22], in the present survey, the overall percentage of Maltese consumers willing to spend more for animal welfare foods remains limited (below 50%), and almost 30% of respondents reported their willingness to pay to be price dependent (Figure 5), unprecedented data in Eurobarometers. The overall distribution across price categories is, however, comparable to the 2023 Eurobarometer data [22]. One exception is a slight increase in consumers willing to spend between 10 and 20% more, reflecting a modest shift toward optimism in this price bracket. This suggests that while price sensitivity has grown, preferences within specific price ranges have not significantly changed.

The strong support for a national animal welfare label (86% of respondents were in favour) reflects clear consumer demand for transparency and standardization in animal welfare claims. Policymakers should act on this widespread approval by developing and implementing a national certification scheme. Clear labelling can build trust and empower consumers to make informed decisions. Marketing efforts should also emphasize the integrity and rigor of such labels to maintain consumer confidence.

The mentioned perception (by 40% of respondents) that there is an insufficient choice of high-welfare products suggests that increasing the visibility and availability of such options could better meet consumer demand. Retailers should explore tiered pricing models to cater to both price-sensitive and more affluent consumers, potentially broadening the market for welfare-friendly products.

When asked what type of information they would like or expect to find on an animal welfare label, most consumers expressed a preference for comprehensive labelling that includes all the mentioned welfare aspects (i.e., farm conditions, transport, feeding practices, antibiotic use, and slaughtering methods). This suggests that most consumers value a holistic view of animal welfare that covers each stage of the production process. A study conducted by the European Commission in 2022 reached similar conclusions [16], even if farm animal welfare is managed and valued differently among the different Member States [39].

Lastly, three-quarters of respondents gave a positive answer when asked if they believed their consumer choices could positively impact the welfare of farm animals, indicating trust in the potential of their purchasing choices to promote an improvement.

### 4.2. Class Distribution Analysis

The results from the Chi-square analysis indicate that business owners show lower support for a national animal welfare label compared to other professional categories. This inconsistency can be interpreted considering that business owners may view labels and certifications as burdensome or costly. In other words, while business owners may value the ethical aspects of animal welfare, they are likely more cautious about measures that could impact their profitability, even when these align with broader consumer sentiment. Differences in perceptions of animal welfare, support for national welfare labels, and the influence of consumer behaviour on animal welfare between urban and rural populations are highlighted in the existing literature [28,29,38,40,41]. Some studies, however, have not found a link between concern for animal welfare and demographic factors such as age, sex, education, or income levels [28,38,41]. Despite this, a general trend in the literature suggests that younger individuals, those with higher education, and those with higher incomes are more likely to express concern for animal welfare [29,37], although the strength of the connection between these variables and attitudes toward animal welfare has not been addressed [36]. Additionally, people with direct exposure to agricultural practices, such as rural residents, often assess farming practices less critically compared to urban populations. This is typically due to their closer connection to the realities of farming, which often leads to a more favourable (or at least less severe) view of farm welfare conditions, even if these conditions do not align with the standards expected by urban residents.

Furthermore, rural populations may also display a certain scepticism toward welfare labels, possibly due to their practical knowledge of the legislation on the protection of farm animals and the perceived disconnection between label effectiveness, fair trade and actual improvements in animal welfare. On the other hand, urban residents, who are more distanced from agricultural practices, tend to place higher demands on the transparency and ethics of production processes [42].

The gender differences in willingness to pay for animal-friendly products observed in this study reflect broader trends seen in Eurobarometer surveys. Women were more likely than men to pay a premium price for these products, particularly when the price increase was modest. This aligns with the 2023 Eurobarometer report on attitudes towards animal welfare [22]. Women also tend to show greater concern for environmental and ethical issues, which likely explains their higher willingness to pay a premium. This is consistent with broader research suggesting that women are generally more focused on sustainability and ethical consumption, influenced by social, cultural, and psychological factors [21,22,33].

Moreover, this gender disparity in willingness to pay is also reflected in studies on food labelling and ethical consumerism, which show that women are more likely to be influenced by information on animal welfare standards when making purchasing decisions [19,22]. While men may prioritize factors like price and convenience more heavily, women are typically more willing to invest in ethical products, suggesting that they may place a higher value on the welfare of animals, even if it requires a small financial sacrifice.

This gender-based difference in purchasing behaviour could be a key factor for policymakers and marketers to consider when designing animal welfare-related campaigns or interventions aimed at encouraging higher consumer engagement with welfare labels and ethical products.

### 4.3. Cluster Analysis

Although respondents displayed high levels of concern and awareness toward animal welfare when answering the individual questions, the cluster analysis did not reveal a clear-cut segmentation of the population into distinct attitudinal groups. The partial overlap between clusters in the Principal Component Analysis projection suggests that attitudes toward animal welfare are quite evenly distributed across the population. This may reflect a broader societal trend, in which growing sensitivity and media attention toward animal welfare issues have contributed to a shared baseline awareness, transcending differences in age, education, or dietary habits [43]. As a result, rather than identifying a specific group of highly engaged individuals, the findings confirm a general convergence of public opinion on the relevance of animal welfare. This homogenization may indicate that animal welfare has become a widely accepted ethical concern, thus reducing the likelihood of identifying sharply divergent consumer segments, as might have been the case in earlier studies.

### 4.4. General Considerations

From a general standpoint, the findings from this study highlight a consumer behaviour paradox that has already been described in the literature about ethical or sustainable behaviour. In short, despite being concerned and having strong ethical beliefs about animal welfare, consumers do not translate their attitudes into corresponding purchasing behaviour. This ethical paradox, also known as “the citizen-consumer disconnect”, has been described, for example, in Irish consumers [28] but seems to also be largely present in Eurobarometer surveys [21,22]. Several reasons may explain this paradox and why it is so prevalent: difficulty processing information, abrogation of responsibility to others, affordability and availability of products, etc. [44]. A “moral blindness” mechanism has also been described, in which the dissonance between the values we express in our words and with our actions can be explained by a willed blindness towards the effects that our meat production and consumption have on animals, the environment and the climate [45]. This blindness, not due to a lack of knowledge or scientific uncertainty, would enable consumers to see themselves as moral beings, despite their lack of action aimed at reducing meat consumption or improving animal welfare levels [45]. Some nudging mechanisms to overcome this discrepancy and foster behavioural change have also been proposed [46] and mostly involve using behavioural mechanisms to promote a change in consumption habits.

Data from this survey substantially confirm what emerged from the most recent Eurobarometers [21,22] and provide additional insights on the relationship between demographic characteristics and some aspects of Maltese consumers’ sensitivity towards animal welfare. Although it was not possible to draw a demographic profile of Maltese consumers concerned about animal welfare, this result, together with the high degree of concern expressed towards animal welfare, suggests that interest might be widespread in the survey population, disentangling from a specific cluster of consumers.

However, in addition to the well-known effects of social desirability bias when ethical questions are posed, one limitation of the present study could be the use of telephone surveys, which are known to present intrinsic limitations such as concentration issues, absence of visual cues, and constraints related to time and accessibility. Future investigations could also consider employing face-to-face surveys and organizing focus groups to overcome these challenges. This approach would allow for a more nuanced understanding of participants’ responses and reduce the impact of bias in phone-based surveys.

## 5. Conclusions

This study provides valuable comprehension into the perceptions and behaviours of Maltese consumers regarding animal welfare in food production.

The findings indicate a significant awareness and concern for animal welfare among respondents, suggesting that ethical considerations are becoming increasingly important in consumer choices. However, this awareness does not translate into actual purchasing behaviour, as the willingness to pay remains limited.

The reliance on mass media as the primary source of information highlights the need for more technically sound and scientifically grounded communication and educational campaigns. These initiatives should aim not only to provide clear and transparent content but, above all, to deliver accurate, validated information that can effectively guide consumers in understanding animal welfare standards and practices.

The strong support for introducing animal welfare labelling and the impact of consumers’ choices on animal welfare reflects a demand for transparency in food production, which can empower consumers to make informed choices.

In conclusion, this study highlights the importance of advancing a culture of animal welfare among Maltese consumers and suggests interesting steps to enhance ethical food production practices. By addressing knowledge gaps, promoting transparency through labelling, and highlighting the connection between consumer choices and animal welfare, stakeholders can significantly improve the treatment of animals within the food supply chain, ultimately aligning production practices with the values of informed consumers.

## Figures and Tables

**Table 1:** Sociodemographic characteristics of the respondents (N = 384).

Class	Percentage
**Gender**	
Female	48.7
Male	50.8
Other	0.3
Prefer not to say	0.3
**Age**	
15–24	23.2
25–39	38.3
40–54	21.4
Over 55	16.7
Prefer not to say	0.5
**Household size**	
1–2	30.0
3–4	57.3
5+	12.8
**Education**	
Non-formal education	1.8
Basic (able to read and write)	14.6
Secondary (obtained O levels)	17.5
Post-Secondary (obtained A levels)	28.1
Tertiary (graduated from university)	38.0
**Employment**	
Employee	67.2
Business owner/freelance	9.9
Retired	6.0
Homemaker	3.7
Unoccupied	5.2
Student	8.1
**Annual household income (€)**	
<10,000	3.4
11,000–20,000	7.8
21,000–35,000	18.5
36,000–50,000	21.1
51,000–75,000	7.8
>75,000	6.0
prefer not to say	35.7
**Area**	
Urban	72.7
Rural	27.3

**Table 2:** Effects of the sociodemographic characteristics on selected answers. Data are expressed as percentages of respondents.

**Question: Would you support the introduction of a national welfare label?**							
	**Occupation**						
**Answer**	**Student**	**Employee**	**Business owner/freelance**	**Retired**	**Homemaker**	**Unoccupied**	**Fisher’s exact test**
**Yes**	87	87	79	78	93	80	G^2^ = 24.7 *p* = 0.006
**No**	0	4	21	9	0	5	
**Don’t know**	13	9	0	13	7	15	
**Question: Perceived level of welfare of farm animals raised in Malta**							
	**Living area**						
**Answer**	**Rural**	**Urban**	**Chi-squared statistic**				
**Don’t know**	8	5	χ^2^ = 12.1 *p* = 0.034				
**Poor**	10	12					
**Just acceptable**	22	32					
**Medium**	33	37					
**Good**	25	13					
**Excellent**	3	1					
**Question: Would you support the introduction of a national welfare label?**							
	**Living area**						
**Answer**	**Rural**	**Urban**	**Chi-squared statistic**				
**Yes**	81	87	χ^2^ = 8.73 *p* = 0.013				
**No**	11	4					
**Don’t know**	8	9					
**Question: Can your consumer choices affect the welfare of farm animals?**							
	**Living area**						
**Answer**	**Rural**	**Urban**	**Fisher’s exact test**				
**Yes, certainly**	25	85	G^2^ = 20.1 *p* < 0.001				
**Yes, probably**	46	145					
**No, probably not**	10	24					
**No, certainly not**	0	9					
**Don’t know**	19	16					
**Willingness to pay more for animal-friendly foods**							
	**Gender**						
**Answer**	**Female**	**Male**	**Chi-squared statistic**				
**No**	19	20	χ^2^ = 36.7 *p* = 0.006				
**Up to 5%**	17	22					
**6–10%**	21	14					
**11–20%**	10	8					
**More than 20%**	4	3					
**Depends on the price**	24	29					
**Don’t know**	5	4					

**Notes to the table:** For ease of interpretation and comparison, data are expressed as percentages of respondents. Chi-square statistics and Fisher’s exact tests were calculated on the number of observations (see the Materials and Methods, Section 2.3). G^2^ = log-likelihood ratio.

## Data Availability

The data presented in this study are available on request from the corresponding author.

## Supplementary Materials

The following supporting information can be downloaded at: https://www.mdpi.com/article/10.3390/ani15111634/s1, Questionnaire text.
