# Supplementary material for: Perception and Awareness of Animal Welfare Among Residents of Malta
Source: Animals (Basel). 2025 Jun 2;15(11):1634. doi: 10.3390/ani15111634 (PMC12153805; doi:10.3390/ani15111634)
Supplement: Supplementary file 1 [file animals-15-01634-s001.zip › animals-3540608-supplementary.pdf]

Animal welfare perception and knowledge among people living in Malta

1. **Code____________**

Demographics and consumption habits

1a. Where do you live? ___________________

1b. Do you live in an urban or in a rural area?

□ Rural area
□ Urban area

2. Sex

□ Male □ Female □ Other □ Prefer not to say

3. Age

□ Age (last birthday) ____________
□ I prefer not to say

4. Level of education:

□ Non formal education
□ Basic (able to read and write)
□ Secondary (obtained O levels)
□ Post-Secondary (obtained A levels)
□ Tertiary (graduated from university)

5. Occupation

□ Employee
□ Business owner/freelance
□ Retired
□ Homemaker
□ Unoccupied
□ Student
□ Other (please specify) ______________

6. How many people are living in your household (including yourself)? _____________________

7. Household net income per year

□ I prefer not to say
□ Below 10,000€
□ Between 11,000€ and 20,000€

□ Between 21,000€ and 35,000€
□ Between 36,000€ and 50,000€
□ Between 51,000€ and 75,000€
□ Over 75,000€

8. Which kind of diet do you follow?

a) Omnivorous (consumption of both vegetal and animal-derived foods)
b) Vegetarian
c) Vegan

9. How often do you eat the following kind of meat/meat products (e.g., salami, hamburgers, fish etc.)?

Meat	At least once a week	More than once a week	At least once a month	Less than once a month	Never
Rabbit	□	□	□	□	□
Chicken	□	□	□	□	□
Pork	□	□	□	□	□
Beef	□	□	□	□	□
Lamb	□	□	□	□	□
Fish	□	□	□	□	□

Knowledge and perception about animal welfare

10. How do you rate your level of knowledge regarding farm animal welfare?

a) Very good
b) Good
c) Medium
d) I just have some basic knowledge
e) No knowledge (if this answer is chosen, the next question must be skipped)

11. Where does your knowledge regarding animal welfare on farm come from?

a) Mass media (Television, web, newspapers)
b) Direct knowledge (1-2 visits to farms)
c) Direct knowledge (more than 2 visits to farms)
d) I am a farmer/veterinarian/agronomist/etc.
e) Other (please specify) _______________

12. In your opinion, which ones, among these aspects, are the most important in determining the level of animal welfare (multiple answer allowed)

a) Sufficient space
b) Adequate transports

c) Expertise of farmers/employees
d) Access outside
e) Natural behaviour
f) Humane slaughtering
g) Absence of mutilation
h) All above
i) None of the above

13. According to your knowledge, which of the following production phase/phases are regulated by laws on animal protection in Malta? (Multiple answer allowed)

a) Rearing
b) Feeding
c) Transport
d) Slaughtering
e) All the above
f) None of the above

14.Please rate on a 1-to-5 scale to the level of welfare you think the following species have on farm (1=poor; 2=just acceptable; 3=medium; 4=good; 5=excellent)

	Don't Know	1	2	3	4	5
Laying hens	☐	☐	☐	☐	☐	☐
Broilers	☐	☐	☐	☐	☐	☐
Pigs	☐	☐	☐	☐	☐	☐
Beef cattle	☐	☐	☐	☐	☐	☐
Dairy cows	☐	☐	☐	☐	☐	☐
Rabbits	☐	☐	☐	☐	☐	☐
Fish	☐	☐	☐	☐	☐	☐

15. Please score, on a 1-5 scale, the importance you attribute to animal welfare at time of food purchasing

1= no importance; 2=little importance; 3=medium importance; 4=high importance; 5=extremely high importance

Score ________

16. In your opinion, what products are obtained respecting high animal welfare standards (multiple answer allowed)

a) Organic products
b) Certified animal welfare labelling
c) Products obtained from extensive farming

d) Products obtained from intensive farming
e) Protected designation of origin products
f) Other (please specify) ________________

17. In your opinion, products obtained respecting high animal welfare standards are also:
(1=completely disagree; 2= partially disagree; 3=neutral; 4=agree; 5= completely agree)

	Don't know	1	2	3	4	5
More safe	□	□	□	□	□	□
More reliable	□	□	□	□	□	□
More tasty	□	□	□	□	□	□
Heathier	□	□	□	□	□	□
More fresh	□	□	□	□	□	□
Greener	□	□	□	□	□	□
More expensive	□	□	□	□	□	□
More ethical (animal protection, fairtrade, sustainability)	□	□	□	□	□	□
Other (please specify_____________________)	□	□	□	□	□	□

18. Please rate the level of welfare you feel animals raised in Malta have
a) Don't know
b) Poor
c) Just acceptable
d) Medium
e) Good
f) Excellent

Animal welfare labelling and purchasing behavior

19. Do you think that in Malta there is currently in markets and supermarkets a sufficient choice of food products obtained from animals having high welfare standards?
a) yes, certainly
b) yes, probably
c) no, probably not
d) no, certainly not
e) don't know

20. Would you be willing to pay more for products sourced from animal welfare-friendly (higher animal welfare level) production systems?
a) No, you are not ready to pay more
b) Yes, you would be ready to pay up to 5% more

c) Yes, you would be ready to pay 6% to 10% more
d) Yes, you would be ready to pay 11% to 20% more
e) Yes, you would be ready to pay more than 20% more
f) It depends on the price of the product
g) Don’t know

21. Currently in Malta, there is not a national label indicating the level of welfare of the animals from which foods intended for humans are obtained. Would you support the idea to introduce one?

a) Yes
b) No (if this answer is chosen, the next question must be skipped)
c) Don’t know

22. What kind of information would you like/expect to find in an animal welfare label?

a) Farm conditions/methods
b) transport
c) slaughtering
d) feeding
e) use of antibiotics
f) expertise of the staff
g) all the above
h) none of the above

23. Do you think your choices as a consumer can positively affect the welfare level of animals intended for animal-derived food production?

a) yes, certainly
b) yes, probably
c) no, probably not
d) no, certainly not
e) don’t know
